# Restoration of Dioxin-Induced Damage to Fetal Steroidogenesis and Gonadotropin Formation by Maternal Co-Treatment with α-Lipoic Acid

**DOI:** 10.1371/journal.pone.0040322

**Published:** 2012-07-20

**Authors:** Takayuki Koga, Takumi Ishida, Tomoki Takeda, Yuji Ishii, Hiroshi Uchi, Kiyomi Tsukimori, Midori Yamamoto, Masaru Himeno, Masutaka Furue, Hideyuki Yamada

**Affiliations:** 1 Graduate School of Pharmaceutical Sciences, Kyushu University, Fukuoka, Japan; 2 Faculty of Pharmaceutical Sciences, Sojo University, Kumamoto, Japan; 3 Research and Clinical Center for Yusho and Dioxin, Kyushu University Hospital, Fukuoka, Japan; 4 Department of Obstetrics, Fukuoka Children’s Hospital, Fukuoka, Japan; 5 Faculty of Pharmaceutical Sciences, Nagasaki International University, Sasebo, Japan; 6 Graduate School of Medical Sciences, Kyushu University, Fukuoka, Japan; Kaohsiung Chang Gung Memorial Hospital, Taiwan

## Abstract

2,3,7,8-Tetrachlorodibenzo-*p*-dioxin (TCDD), an endocrine disruptor, causes reproductive and developmental toxic effects in pups following maternal exposure in a number of animal models. Our previous studies have demonstrated that TCDD imprints sexual immaturity by suppressing the expression of fetal pituitary gonadotropins, the regulators of gonadal steroidogenesis. In the present study, we discovered that all TCDD-produced damage to fetal production of pituitary gonadotropins as well as testicular steroidogenesis can be repaired by co-treating pregnant rats with α-lipoic acid (LA), an obligate co-factor for intermediary metabolism including energy production. While LA also acts as an anti-oxidant, other anti-oxidants; *i.e.*, ascorbic acid, butylated hydroxyanisole and edaravone, failed to exhibit any beneficial effects. Neither wasting syndrome nor CYP1A1 induction in the fetal brain caused through the activation of aryl hydrocarbon receptor (AhR) could be attenuated by LA. These lines of evidence suggest that oxidative stress makes only a minor contribution to the TCDD-induced disorder of fetal steroidogenesis, and LA has a restorative effect by targeting on mechanism(s) other than AhR activation. Following a metabolomic analysis, it was found that TCDD caused a more marked change in the hypothalamus, a pituitary regulator, than in the pituitary itself. Although the components of the tricarboxylic acid cycle and the ATP content of the fetal hypothalamus were significantly changed by TCDD, all these changes were again rectified by exogenous LA. We also provided evidence that the fetal hypothalamic content of endogenous LA is significantly reduced following maternal exposure to TCDD. Thus, the data obtained strongly suggest that TCDD reduces the expression of fetal pituitary gonadotropins to imprint sexual immaturity or disturb development by suppressing the level of LA, one of the key players serving energy production.

## Introduction

Dioxins, such as 2,3,7,8-tetrachrolodibenzo-*p*-dioxin (TCDD), are widespread and persistent toxic environmental pollutants, and their harmful effects are of great concern. Dioxins induce toxic effects on laboratory animal such as wasting syndrome, tumorigenicity and immunotoxicity, and the mechanisms are believed to involve the activation of the aryl hydrocarbon receptor (AhR) [1]–[4]. In addition, when pregnant animals are exposed to TCDD, a number of disorders occur in the fetuses and newborn pups [5], [6]. Such toxic effects on later generations are more serious, because they are produced by TCDD at lower doses and only have a weak effect on the dams [6], [7]. The pup disorders produced by in utero and lactational exposure to TCDD include growth retardation of reproductive function, abnormalities in sexual behavior and a reduction in spermatogenesis [6]. The mechanism underlying the above disorders produced by TCDD may be related to estrogenic/anti-estrogenic effects [8], [9], regulation of sex-steroid metabolism [10], and an increase in the proteosomal degradation of receptors [11], [12]. Although these mechanisms and their combinations may partially explain the reproductive and developmental toxicity caused by TCDD, it is not yet fully understood how these mechanisms contribute to the toxicity.

Our laboratory has found that treating pregnant rats with TCDD reduces expression of fetal testicular proteins necessary for sex-steroid biosynthesis [13], [14]. The reduced proteins include steroidogenic acute-regulatory protein (StAR) and cytochrome P450 (CYP) 17: StAR is a cholesterol transporter facilitating the rate-limiting step of the production of steroid hormones and CYP17 is one of the key enzymes involved in sex-steroid synthesis. The reduced expression of these proteins was observed following treatment of pregnant rats with more than 1 µg/kg TCDD on gestational day (GD) 8 and 15 [13]. Further evidence has suggested that the damage produced by TCDD on the fetal expression of pituitary gonadotropins, luteinizing hormone (LH) and follicle-stimulating hormone (FSH), is a pre-requisite of the down-regulation of steroidogenic proteins [13], [14]. In accordance with this, a TCDD-produced reduction in the expression of both gonadotropins and steroidogenic proteins concomitantly occurs at late fetal ages, and disappears shortly after birth [13]. More importantly, our recent study has shown that the abnormal sexual behavior in the pups born from TCDD-exposed dams is attributable to the suppression of LH/FSH expression during the fetal stages [15]. This is based on the evidence that the administration of equine chorionic gonadotropin (eCG), an LH-mimicking hormone, to TCDD-exposed fetuses restored not only the reduced expression of fetal testicular steroidogenic proteins but also impaired sexual behavior at adulthood [15].

TCDD and related substances enhance the production of reactive oxygen species (ROS) in many organs, and this is considered to be one of the mechanisms underlying the chronic as well as acute toxicity of dioxins [16]–[19]. Also, in some forms of the reproductive toxicity produced by TCDD, enhanced ROS production has been suggested to play a role [20]–[22]. In relation to this, anti-oxidants combat an LH reduction produced by Aroclor 1254, a mixture of polychlorinated biphenyls, in adult mice [23]. However, it is unclear whether TCDD reduces the expression of pituitary LH in the fetuses by enhancing oxidative stress. To address this issue, we firstly examined the effects of four anti-oxidants on a TCDD-produced reduction in the fetal expression of steroidogenic proteins and gonadotropins. The anti-oxidants examined were two endogenous substances [α-lipoic acid (LA) and ascorbic acid (VC)], a drug acting as a radical scavenger (edaravone), and a classical anti-oxidant [3-*tert*-butyl-4-hydroxyanisole (BHA)]. During the course of the present study, we observed that only LA has a beneficial effect. Since LA acts as an endogenous cofactor for intermediary metabolism as well as an anti-oxidant, we then conducted a metabolomic study to clarify the mechanism whereby LA restores TCDD-induced effects on the fetal pituitary-gonad axis.

## Materials and Methods

### Materials

TCDD was purchased from Accu Standard Inc. (New Haven, CT). The oxidized form of racemic LA was obtained from Nacalai Tesque (Kyoto, Japan). Edaravone was a kind gift from Mitsubishi Tanabe Pharma Corporation (Osaka, Japan). The other reagents were of the highest grade commercially available.

**Figure 1:**
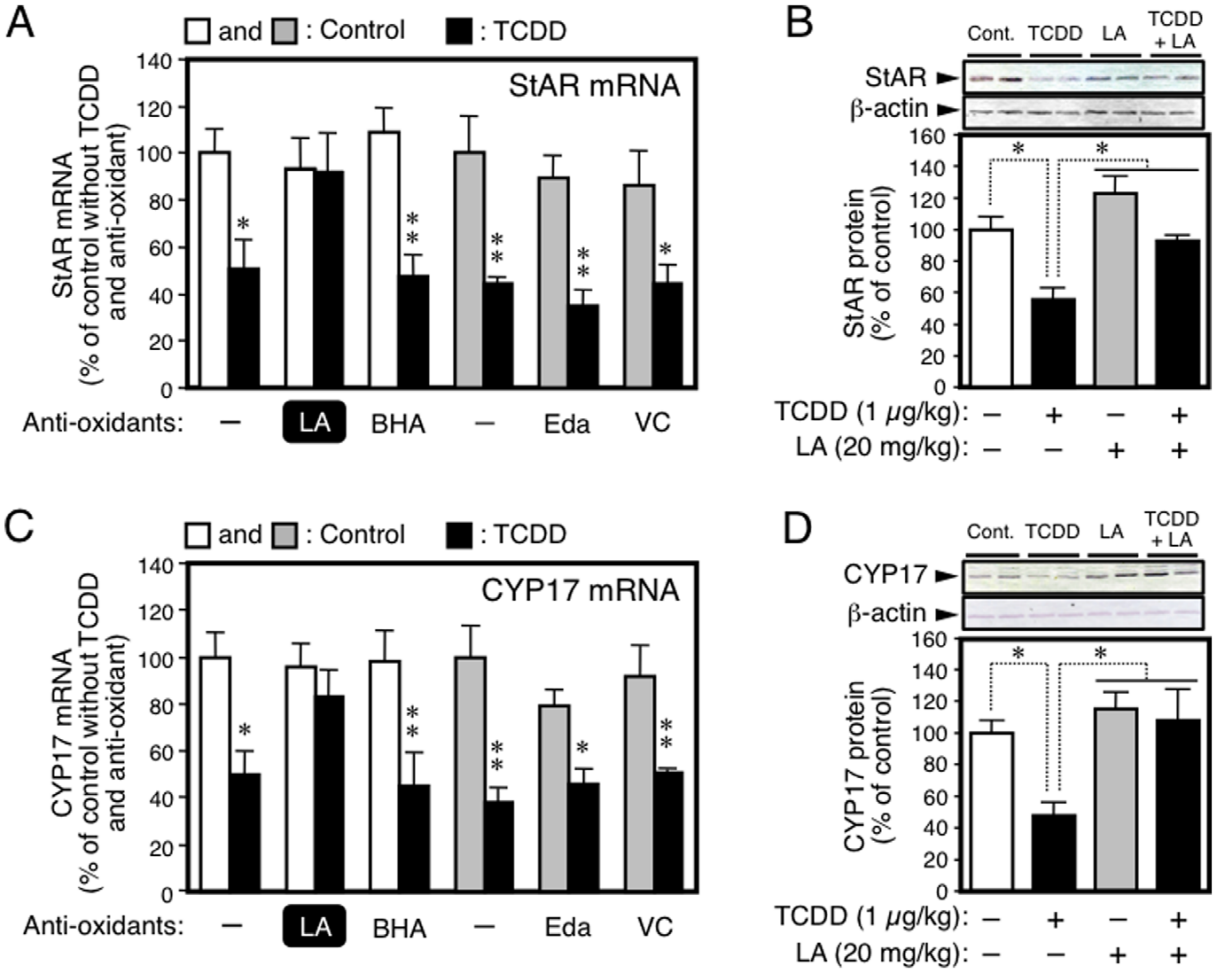
The LA-specific recovery from a TCDD-induced reduction in the fetal expression of testicular StAR and CYP17; A, StAR mRNA; B, StAR protein; C, CYP17 mRNA; and D, CYP17 protein. The fetal (GD20) testis was analyzed after maternal exposure to TCDD (GD15) and anti-oxidants (GD15-20). The expression of mRNA and protein was analyzed RT-PCR and immunoblotting, respectively. See Materials and Methods for the details of animal treatment and analytical methods. Edaravone is abbreviated as Eda. The levels of StAR and CYP17 mRNAs were normalized by β-actin mRNA. In the white bar control, pregnant rats were treated with DMSO alone or anti-oxidant dissolved in DMSO. In the shaded bar control, dams were given aqueous NaCl alone or anti-oxidant dissolved in this solution. Each bar represents the mean value relative to the control ± SEM of 10 fetuses, each 2 of which were removed from 5 different dams. In processing the data, the values of two fetuses from one dam were averaged to become one analytical unit. Thus, the data are shown as N = 5 dams. *p<0.05 and **p<0.01, from the respective controls (panels A and C) or between a pair indicated (panels B and D).

**Figure 2:**
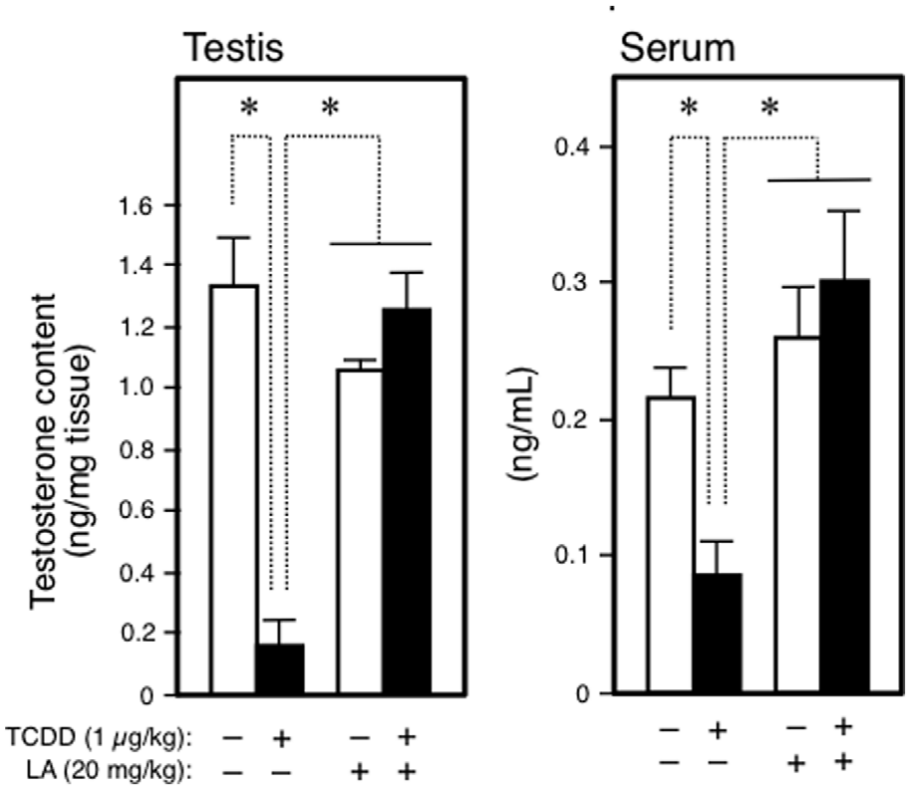
Reduction by TCDD in the fetal content of testosterone and its recovery by LA. The testes and sera of GD20 fetuses born from the dams treated with TCDD at GD15 were analyzed by LC-TOF-MS. Each bar represents the mean ± SEM (N = 3–5 dams). *p<0.05 between a pair indicated.

### Animals and Treatments

All experiments were approved by the Institutional Animal Care and Experiment Committee of Kyushu University (approval No: A23-013-0). Female Wistar rats (7-week-old) and male Wistar rats (6- or 9-week-old) were purchased from Kyudo Co. Ltd. (Kumamoto, Japan). In some experiments, female rats were paired overnight with male rats. The next morning, sperm in the vaginal smears were checked using a microscope to confirm pregnancy. When sperm was detected, the day was designated as GD0 of pregnancy. Pregnant rats at GD15 were given intravenously one of the following anti-oxidants: LA, 1, 5, 10 or 20 mg/kg/200 µl dimethyl sulfoxide (DMSO); BHA, 500 mg/kg/200 µl DMSO; edaravone, 3 mg/kg/200 µl aqueous NaCl; and VC, 200 mg/kg/200 µl aqueous NaCl. Edaravone and ascorbic acid were dissolved in 1 M NaOH, the pH of the solution was adjusted to 7.4 with 1 M HCl, and then the mixture was made up to the desired concentration with water. The doses of anti-oxidants were determined by referring to previous studies which reported the placental transfer and brain distribution [24]–[29]. TCDD was given orally to rats at a dose of 1 µg/kg/2 ml corn oil 30 min after the first treatment with an anti-oxidant. The same anti-oxidant given as the first treatment was then given intravenously once a day at the same dose for the next 5 days (GD16-20). Control animals received vehicle alone (DMSO or aqueous NaCl, and corn oil). The fetuses were removed 30 min after the last treatment with anti-oxidant for the analysis of steroidogenic proteins and gonadotropins, mRNA and protein levels.

**Figure 3:**
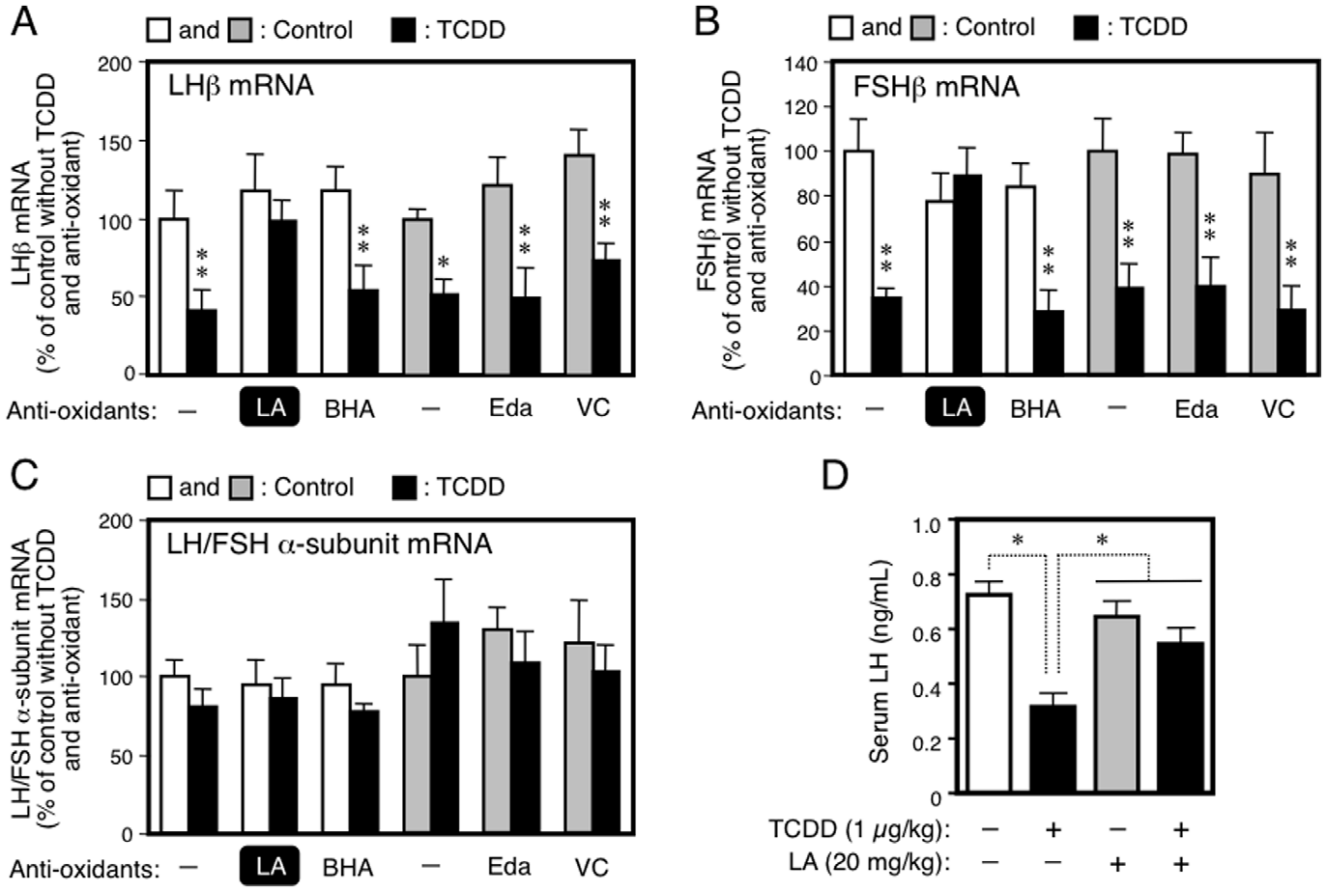
The LA-specific recovery from a TCDD-induced reduction in the fetal expression of gonadotropins: A–C, pituitary levels of LHβ, FSHβ and LH/FSH α-subunit mRNAs, respectively; and D, serum content of LH. Gonadotropin mRNAs in the fetuses (GD20), the parents of which were treated with TCDD (GD15) and anti-oxidants (GD15-20), were determined by RT-PCR. The level of gonadotropin mRNA was normalized by β-actin mRNA. Serum LH (GD20 fetuses) was determined by ELISA. In the white bar control, pregnant rats were treated with DMSO alone or anti-oxidant dissolved in DMSO. In the shaded bar control, dams were given aqueous NaCl alone or anti-oxidant dissolved in this solution. The pituitaries and sera of all male fetuses in one dam were pooled to become one analytical unit. Each bar represents the mean value relative to the control ± SEM of 5 (panels A-C) or 7 (panel D) dams. *p<0.05 and **p<0.01, from the respective controls (A-C) or between a pair indicated (*D*).

**Figure 4:**
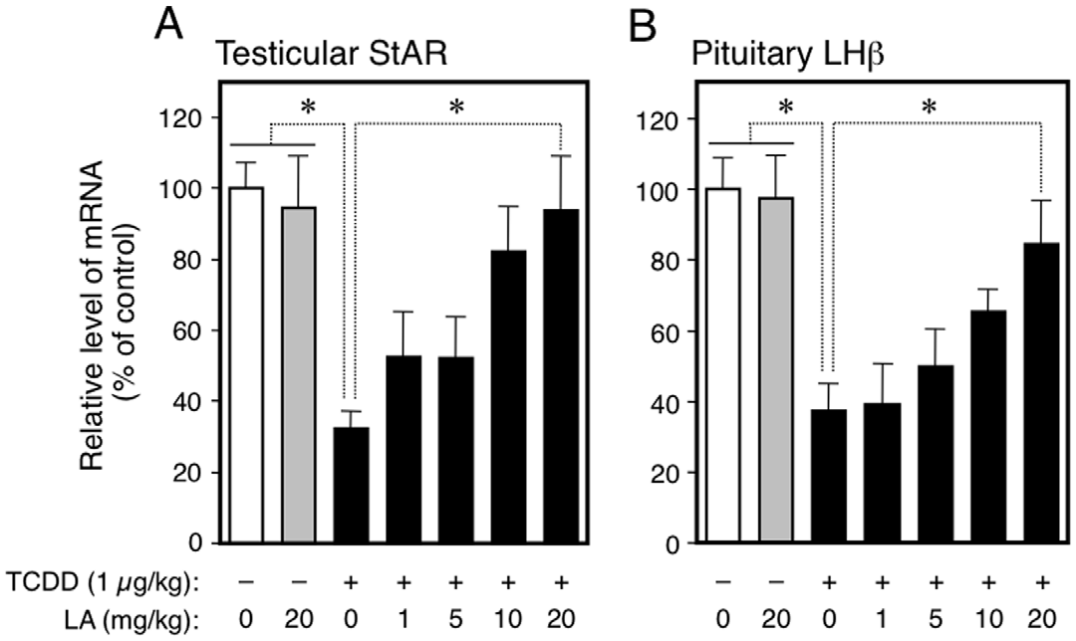
Dose-effect relationship for LA-assisted recovery from a TCDD-induced reduction in the fetal expression of mRNAs coding testicular StAR (A) and pituitary LHβ (B). Testicular StAR and pituitary LHβ mRNAs in the fetuses (GD20) were analyzed after maternal exposure to TCDD (GD15) and LA (GD15-20) by RT-PCR. LA was given to dams at different doses indicated in the figure. See also the legends to Figs. 1 (StAR analysis) and 3 (LHβ analysis) for the details of data processing. In Experiment (A), each bar represents the mean ± SEM of 6–10 fetuses each 2 of which were removed from different dams. In (B), each bar represents the mean ± S.E. of 3–5 pooled fetal pituitaries which were removed from different dams. *p<0.05, comparison between groups indicated.

In a separate experiment, the effect of LA on the acute toxicity of TCDD was examined in pubertal rats. In this case, 6-week-old male Wistar rats were treated intravenously with LA (20 or 40 mg/kg/200 µl DMSO) and then, 30 min later, TCDD (30 µg/kg/2 ml corn oil, orally) was given to the animals. The same dose of LA as that of the first treatment was given once a day for the next 5 days. The organs were removed 30 min after the last treatment with LA and weighed. The liver was homogenized in ten volumes of 1.15% KCl, and centrifuged at 9000×g for 20 min to prepare supernatants as a source of CYPs. All prepared samples were stored at −80°C until use.

### Reverse Transcription-polymerase Chain Reaction (RT-PCR)

The extraction of mRNA and its reverse transcription to cDNA was performed according to the methods reported previously [30]. Primer designs and PCR conditions guaranteeing cDNA amount-dependent amplification for mRNAs coding for gonadotropins, steroidogenic proteins and CYP1A1 were described elsewhere [13], [15].

**Figure 5:**
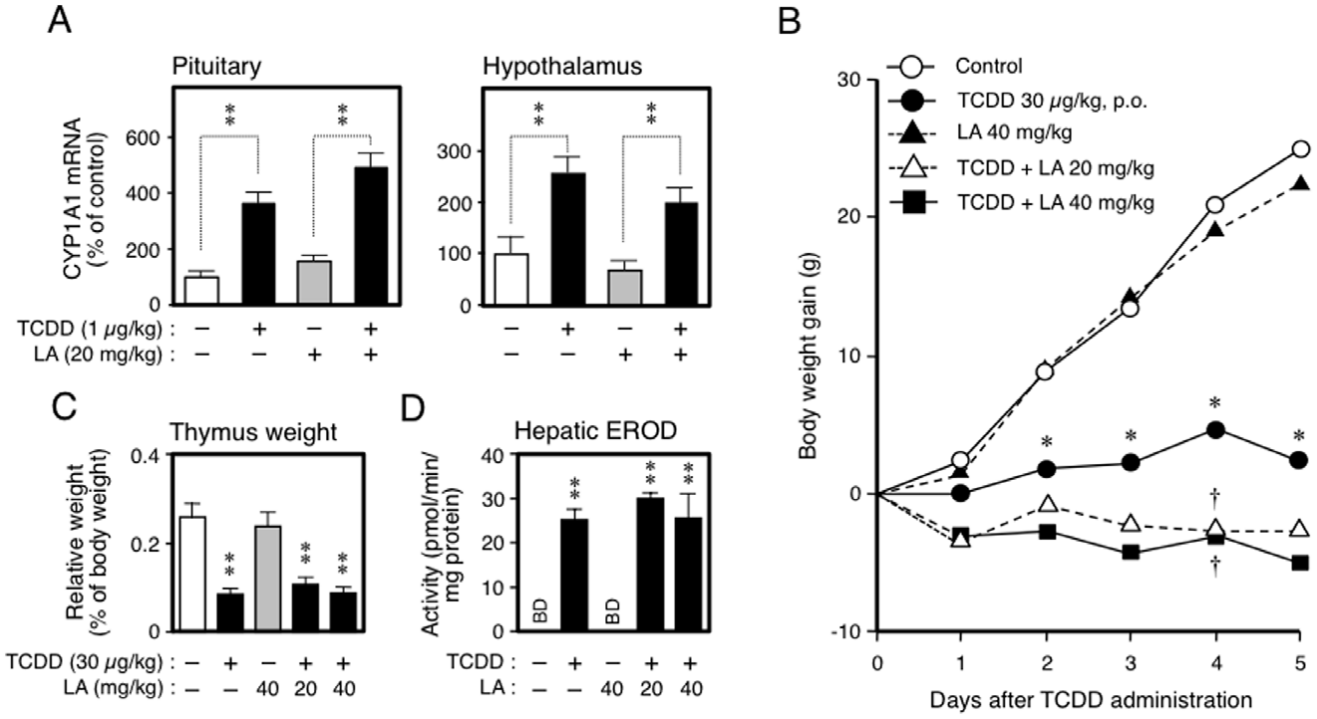
The effects of LA on the induction of fetal brain CYP1A1 and on the acute toxicity of TCDD in pubertal rats. A, Pituitary and hypothalamic CYP1A1 mRNAs in the fetuses (GD20), the parents of which were treated with TCDD (GD15) and LA (GD15-20). B-D, Change in body weight (B), thymic weight (C) and hepatic EROD activity (D) of male pubertal rats treated orally with TCDD (Day 0) and intravenously with LA (from Day 0 to Day 5). In Experiment (*B*), the initial body weights of rats in the control, TCDD, LA, and TCDD + LA-treated groups were 219±6, 218±6, 216±7, 216±8 (LA: 20 mg/kg), and 217±5 (LA: 40 mg/kg) g, respectively. The thymus and liver were removed 30 min after the last administration of LA or vehicle. Each bar or plot represents the mean ± SEM (N = 5 or 6). To avoid confusion, the error bar was omitted from panel *B*. *p<0.05 and **P<0.01, compared with the control, and †P<0.05 compared with the TCDD-treated group. B.D.: below the determination limit (4 pmol/min/mg protein).

### Immunoblotting

Immunoblotting was carried out according to the procedures described elsewhere [15]. The modification introduced was that a fetal testis was homogenized in 15 µl 100 mM potassium phosphate buffer (pH 7.4) containing 0.25 M sucrose, 0.5 mM ethylenediamine tetraacetic acid, 1 mM dithiothreitol, 1 mM phenylmethylsulfonyl fluoride and 20% (v/v) glycerol. The mitochondrial fraction was used for the immunoblotting of StAR and β-actin, and the 9000×g supernatant was used for CYP17 and β-actin. Immunoblotting with anti-StAR, -CYP17 and -β-actin antibodies was carried out by the methods described elsewhere [15]. Antibodies against human StAR was purchased from Santa Cruz Biotechnology Inc. (Santa Cruz, CA). Anti-guinea pig CYP17 antibody was a kind gift from Dr. S. Kominami (Hiroshima University, Hiroshima, Japan).

### Testosterone Determination

The testes and sera of all male fetuses in one dam were pooled, and the combined testes were homogenized with 4-volume of phosphate-buffered saline (PBS). Fetal testis homogenate and serum (each 120 µl) were treated at 60°C for 1 hr in the presence of 1M NaOH to improve the recovery of testosterone. After neutralization with 5M HCl (35 µl) and then NaHCO_3_ (50 mg), the mixture was voltex-mixed for 1 min with 360 µl *n*-hexane-ethyl acetate ( = 3∶2, v/v), centrifuged at 3,000 rpm for 10 min, and the organic layer was collected. The extraction was repeated two more times, and the pooled organic layer was evaporated under a stream of nitrogen. The residue was finally reconstituted with 50 µl MeOH, and an aliquot (10 µl) of the solution was subjected to analysis by high-performance liquid chromatography coupled with time-of-fight mass spectrometry (HPLC-TOF-MS; see the Methods S1 for the conditions). Testosterone was detected at a retention time of 11.2 min by monitoring its molecular ion [base peak, *m/z* 289.2168 ([M+H]^+^]. The calibration curve was constructed by adding testosterone-d_3_ to the target tissue, and its linearity was confirmed over the ranges: 0.17–2.8 ng/mg testis and 0.07–1.1 pg/ml serum.

**Figure 6:**
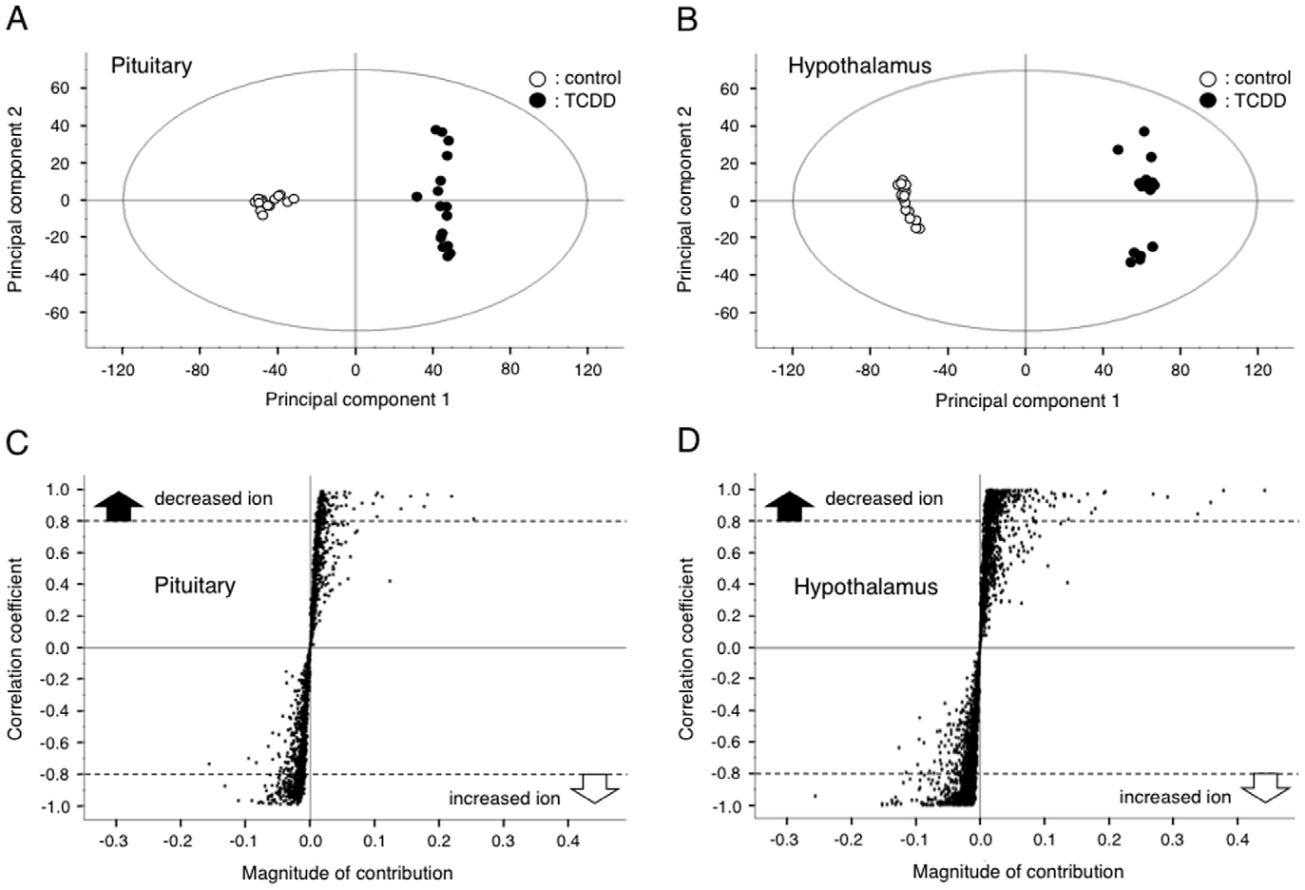
Principal component analysis (PCA) regarding the effect of TCDD on the fetal pituitary and hypothalamic metabolome: the data from negative ion mode analysis. A and B, TCDD effect on the profile of pituitary (A) and hypothalamic (B) metabolome. Each dot is a different animal (N = 16 dams/group). C and D, Fragment ions in LC-TOF-MS analysis that exhibit an alteration following TCDD treatment in the pituitary (C) and hypothalamus (D). Each dot shows a single ion with a particular mass (*m/z*). The criteria for selecting ions which were significantly changed by TCDD was set either at more than 0.8 or less than −0.8 of the correlation coefficient. See Experimental Procedures for details.

**Table 1:** Fetal brain components showing a significant increase or decrease by maternal exposure to TCDD.

	Increased by >2-times	<2-times	Decreased to <1/2 level	>1/2 level
**Hypothalamus**				
	Tyrosine	4-Sphingenin	Stearic acid	Lauric acid
	Phenylalanine	Sphinganin	Lactobionic acid	Tridecanoic acid
	N-Acetylaspartic acid	Taurine	Chenodeoxycholic acid	Myristic acid
	Cortisol	Glutathione	Cholic acid	Valine
	**Isocitric acid**	AMP	4-Hydroxyretinoic acid	Cystamine
	**α-Ketoglutaric acid**	Uridine	Tryptophan	Pipecolic acid
	**Oxaloacetic acid**		Alanine	Thymidine
			**Citric acid**	Adenosine
			***cis*** **-Aconitic acid**	7-Dehydrodesmosterol
			**Fumaric acid**	Calcitriol
				GABA
				Glutamine
				**Succinic acid**
**Pituitary**				
	None	AMP	Glutathione	Uridine
		CMP	Itaconic acid	Valine
		Tryptophan	Ascorbic acid	Threonine
			Palmitoleic acid	Glutamine
			Stearic acid	Oleic acid
				Arachidonic acid

The representative components that were shown by PCA to be significantly changed by TCDD are listed. The data from both positive and negative ion modes were combined. The components of the TCA cycle are shown in bold character.

**Figure 7:**
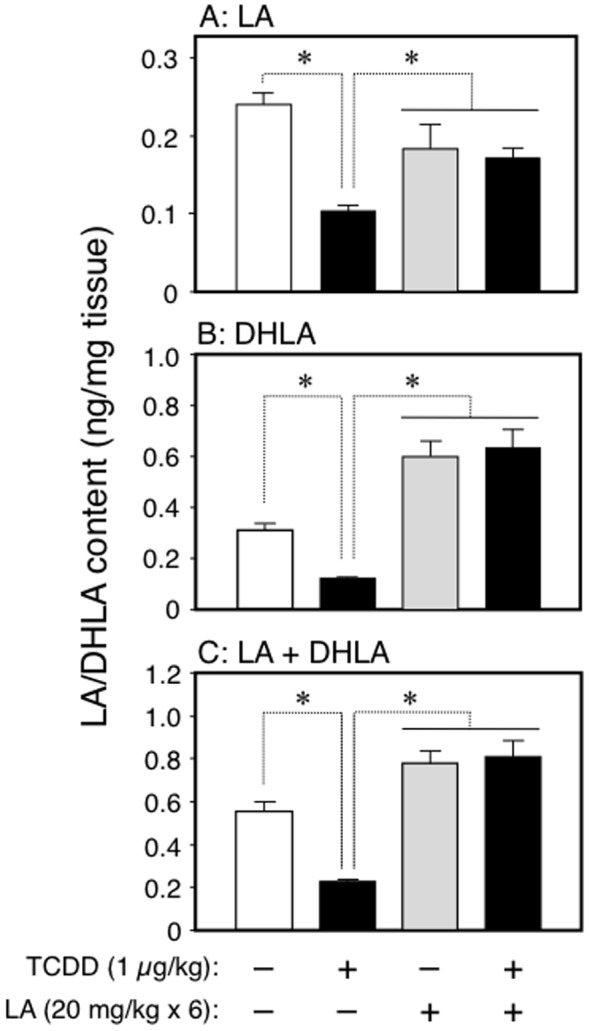
Effect of maternal exposure to TCDD on the fetal content of hypothalamic LA. A, oxidized form of LA; B, reduced form (DHLA) of LA; and C, total content of LA + DHLA. Each bar represents the mean ± S.D. of 5 dams: the hypothalami of male fetuses in one dam were pooled for assay. *Significantly different between the pair indicated (P<0.05).

### Metabolomics and Analysis of Tricarboxylic Acid (TCA) Cycle Components

A change in the metabolomic profile and TCA cycle components was analyzed by the procedures described in the Methods S1. In brief, the hypothalami and pituitaries of fetuses (GD20) were removed from their dams treated with TCDD at GD15. The tissue components were extracted with aqueous methanol, and analyzed by UPLC-TOF-MS. In metabolomics, difference in profile between groups was estimated by multivariate analysis which is also shown in the Methods S1.

### Analysis of LA/dihydrolipoic Acid (DHLA) Content

A change in the content of LA and DHLA, a reduced form of LA, was analyzed by the procedures described in the Methods S1. In brief, the hypothalami of fetuses (GD20) were removed from their dams treated with TCDD at GD15 and with LA at GD15-20. Hypothalamic LA and DHLA were extracted with acetonitrile, and determined by UPLC-TOF-MS.

### Statistical Analysis

The data for the fetuses in one dam were averaged to become an analytical unit, or the data were obtained after combining fetal samples in one dam. Therefore, the variance of data (S.E.) was calculated on the basis of the number of dams. Statistical differences were determined by either Student’s *t*-test for the comparison of two groups or ANOVA with a *post hoc* test using Tukey-Kramer procedures for the comparison of more than two groups.

**Figure 8:**
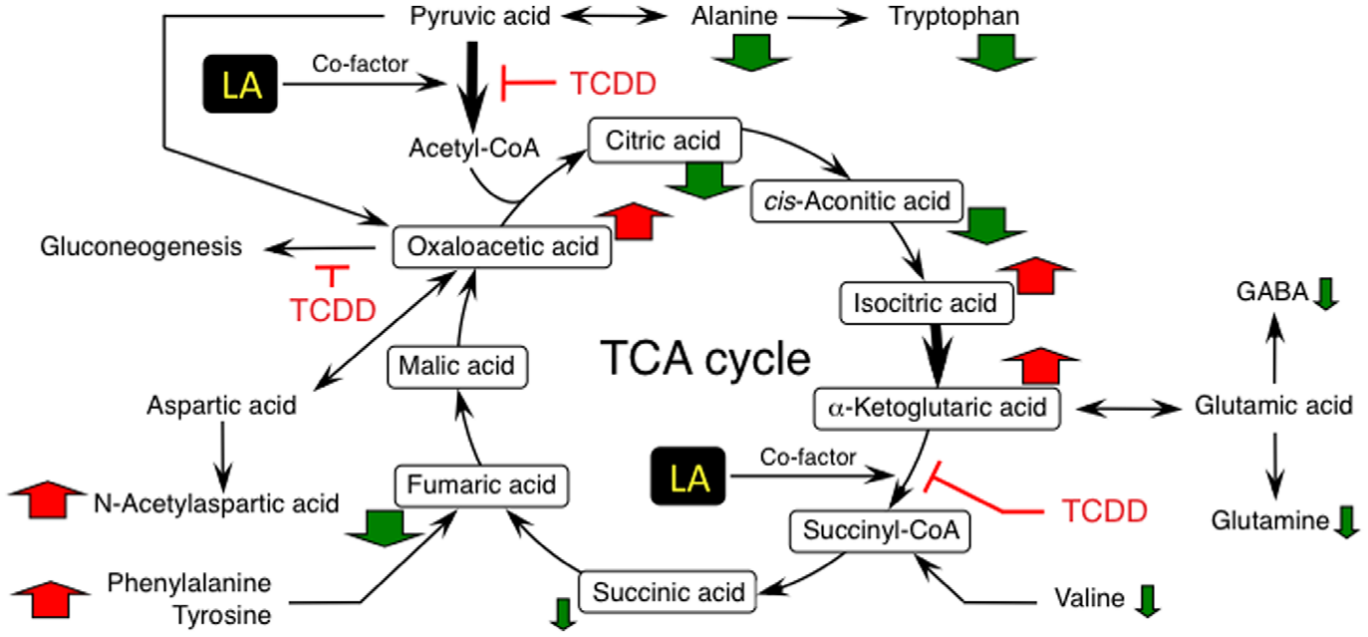
Summary of TCDD effects on fetal hypothalamic changes in the TCA cycle and amino acid metabolism. The upward (red) and downward (green) arrows represent a significant increase and reduction produced by maternal exposure to TCDD. Thick arrows indicate a two-fold or more increase, or a reduction to 1/2 or less, compared with the control. The black thick arrows show the steps which are targets for the regulation of the TCA cycle. Regarding gluconeogenesis, dioxins have been reported to down-regulate phosphoenolpyruvate carboxykinase, a key enzyme involved in this process [46].

**Table 2:** The effect of LA on TCDD-produced alterations in fetal contents of hypothalamic ATP and TCA cycle components.

	Hypothalamic content (pmol/mg tissue) [% of control]							
Component	Control		TCDD		LA		TCDD + LA	
Citric acid	10.9±1.2 [100]		4.87±0.50* [45]		8.13±0.30 [75]		8.49±0.77† [78]	
*cis*-Aconitic acid		0.209±0.033 [100]		0.098±0.016* [47]		0.170±0.014 [81]		0.164±0.008 [78]
Isocitric acid		1.14±0.12 [100]		11.7±0.7* [1,020]		2.52±0.14 [220]		1.19±0.04† [104]
α-Ketoglutaric acid		0.519±0.094 [100]		26.6±0.4* [5,120]		0.871±0.052 [168]		0.165±0.043† [32]
Succinic acid		64.8±8.3 [100]		38.9±4.3* [60]		60.5±5.1 [93]		56.9±2.1 [87]
Fumaric acid		2.34±0.71 [100]		0.781±0.101* [34]		1.12±0.22 [47]		1.64±0.34† [70]
Malic acid		2.68±0.96 [100]		1.76±0.27 [65]		2.27±0.18 [84]		1.44±0.53 [54]
Oxaloacetic acid		8.69±0.98 [100]		43.3±11.0* [498]		15.0±1.6 [173]		8.08±2.02† [93]
ATP		2.19±0.08 [100]		1.01±0.312* [46]		3.68±0.07* [168]		3.07±0.52† [140]

Each value represents the mean ± S.E.M. (N = 5 dams): the hypothalami of male fetuses in one dam were pooled for assay. Significantly different from the control (*) and from the TCDD-treated group (†): P<0.05.

### Other Methods

The fetal serum LH was determined by enzyme-linked immunosorbent assay (ELISA) using a commercial kit (Endocrine Technologies Inc., Newark, CA). The ATP content in the 1,000×g supernatant of fetal hypothalamus was determined using a luciferin/luciferase system (Promega, Madison, WI). The hepatic activity of ethoxyresorufin *O*-deethylase (EROD) was measured by the method of Burke and Mayer [31]. The hypothalamic content of thiobarbituric acid-reactive substances (TBARS) was measured by the method of Ohkawa et al. [32].

## Results

### Effects of Anti-oxidants on Fetal Steroidogenesis

To examine the effects of anti-oxidants on fetal steroidogenesis, pregnant rats at GD15 were treated with an anti-oxidant and TCDD. Following treatment of the dams for 5 days with an anti-oxidant, the expression of StAR and CYP17 in the fetal testis was analyzed. Maternal exposure to TCDD reduced StAR and CYP17 expression in both mRNA and protein levels (Fig. 1, A–D). Co-treatment with LA restored these reductions to the respective control level. However, the other three anti-oxidants, BHA, VC and edaravone, did not produce any recovery of the reduction in these mRNAs (Fig. 1, A and C). A similar picture was also seen for the expression of other steroidogenic proteins. For example, the fetal expression of testicular CYP11A1 and 3β-hydroxysteroid dehydrogenase mRNAs was attenuated by maternal treatment with TCDD, and only LA restored these reductions (Figure S1, A and B). In accordance with a reduction in the expression of steroidogenic proteins, TCDD markedly reduced the testicular and serum contents of testosterone in fetuses (Fig. 2). Also in this case, maternal co-treatment with LA repaired the disorder to the control situation. Although TCDD damaged the steroidogenesis in the fetal testis, this tissue exhibited little pathological change (hemayoxylin/eosin staining) even after TCDD and TCDD + LA treatments (data not shown). This seems to relate to the previous suggestion that TCDD action on the fetal steroidogenesis is due to a mechanism different from direct damage to the testis [13], [15].

### Effects of Anti-oxidants on Fetal Gonadotropins

We then focused on pituitary gonadotropins, the upstream regulators of testicular steroidogenesis. As LH and FSH consist of two subunits, a common α-subunit and a specific β-subunit, we analyzed the expression of both subunits. Like the testicular steroidogenic proteins, TCDD given to the dams reduced LHβ and FSHβ mRNAs in the fetal pituitary (Fig. 3, A and B). Only LA successfully reversed the TCDD-produced damage while other anti-oxidants failed to exert the same effect (Fig. 3, A and B). The fetal expression of LH/FSH α-subunit mRNA did not change following treatments of the dams with TCDD and any anti-oxidant (Fig. 3C). Prolactin, another gonadotropin, was also resistant to TCDD treatment (Figure S2). In parallel with the change in LHβ mRNA level, the serum concentration of fetal LH determined by ELISA was reduced by maternal exposure to TCDD, and LA restored it (Fig. 3D). To examine the dose-dependence of LA-assisted restoration in steroidogenesis and gonadotropin formation, we administered this substance at five doses to pregnant rats (GD15-20) together with TCDD (GD15). As shown in Fig. 4, LA restored a reduction in the fetal expression of testicular StAR as well as pituitary LHβ mRNAs in a dose-dependent manner. The restoration to the control level was achieved at the maximal dose of 20 mg/kg.

### The Role of AhR-signaling on the Effect of LA

To identify the mechanism of recovery by LA, we then examined the effect of this substance on CYP1A1 induction which is a sensitive marker of the TCDD-dependent activation of AhR. If LA exerts its restorative effect on gonadotropins via the modification of AhR-signaling, it would be expected that the TCDD-produced induction of brain CYP1A1 would also be modified by co-treatment with LA. Because the pituitary formation of gonadotropins is regulated by the hypothalamus, the effect of LA on CYP1A1 induction was examined in these two areas. As shown in Fig. 5A, TCDD increased CYP1A1 mRNA expression in both the fetal pituitary and hypothalamus, indicating that TCDD given to dams reached these areas of the fetuses to induce CYP1A1. However, LA did not change CYP1A1 induction by TCDD in both brain regions. It is of interest to examine whether LA is also an effective protector against the acute toxicity of TCDD. We then focused on this issue, and examined the effect of LA on TCDD-induced wasting syndrome in male pubertal rats. When 30**µg/kg TCDD was given to male pubertal rats, it reduced the body weight gain and thymus weight (Fig. 5, B and C). LA, even at 40 mg/kg, showed no protective effect on these disorders. Conversely, LA at both doses aggravated significantly a TCDD-produced reduction in body weight (Fig. 5B). In agreement with the absence of an LA effect on CYP1A1 induction in the brain (see Fig. 5A), LA did not affect a TCDD-produced increase in hepatic EROD activity which is mediated by CYPs including CYP1A1 (Fig. 5D). The above pieces of evidence suggest that LA has little or no ability to combat the acute TCDD toxicity which is produced by the activation of AhR.

### Effect of LA on TCDD-induced Change in Brain Metabolome

Besides being an anti-oxidant, LA is an obligate co-factor for intermediary metabolism, such as acetyl-CoA production from pyruvic acid and succinyl-CoA production from α-ketoglutaric acid [33], [34]. These two reactions are essential for energy production in the TCA cycle. Thus, it is conceivable that TCDD reduces the expression of fetal LH by affecting the level of the pituitary components necessary for energy production and signal transduction. It is also of interest whether LA can counteract the TCDD effect on the metabolome. To address these issues, we carried out a metabolomic analysis using LC-TOF-MS. As the hypothalamus regulates pituitary function, metabolomic analysis was also conducted in this region. The cellular components were analyzed both in positive ion and negative ion modes. The PCA of the LC-TOF-MS data showed that maternal exposure to TCDD (1 µg/kg) at GD15 altered the metabolomic profile in the pituitary as well as the hypothalamus of GD20 fetuses (Fig. 6, A and B; negative ion mode). However, as can be seen in the 'S-plot', the number of ions, the levels of which were significantly changed by TCDD, contributing to a change in the metabolome profile was more abundant in the hypothalamus than in the pituitary (Fig. 6, C and D). The same profiles were also obtained in the positive ion mode data (Figure S3, A–D). Referring the ion information about mass and retention time to the databases suggests a number of substances which are affected by TCDD (Table 1). The number of altered components was much greater in the hypothalamus compared with the pituitary. In addition, the nature of the substances showing a change was very different between tissues. For example, the components of the TCA cycle were reduced only in the hypothalamus (Table 1, bold character). The quantification of TCA cycle components based on calibration with standards showed that the hypothalamic contents of almost all components were significantly changed following TCDD treatment (Table 2). Especially, oxaloacetic acid, isocitric acid, and α-ketoglutaric acid accumulated 5 times or more compared with the control. These changes were restored or alleviated by co-treatment with LA (Table 2). In agreement with the abnormality in the TCA cycle, the fetal content of hypothalamic ATP was reduced by TCDD. Also in this case, LA again restored the change to the control level (Table 2). Conceivably, the content of endogenous LA in the fetal hypothalamus may be reduced by TCDD and returned to the normal level by the administration of exogenous LA. This was the case, and UPLC-TOF-MS analysis under different conditions from metabolomics revealed that the hypothalamic contents of fetal LA and its reduced form, dihydro-LA (DHLA), were significantly reduced by maternal exposure to TCDD (Fig. 7). The reduced state produced by TCDD was recovered to near the control level or more by treatment with exogenous LA (Fig. 7).

## Discussion

This study provides evidence that LA has the ability to restore a TCDD-induced reduction in the expression of fetal testicular steroidogenic proteins, whereas other anti-oxidants, BHA, VC and edaravone, lack such an effect. Furthermore, the data obtained strongly suggest that LA exerts the above effect through recovery from the TCDD-reduced expression of pituitary gonadotropins. Conceivably, the reason why three anti-oxidants other than LA are ineffective may be their poor distribution to the fetal brain. However, not only LA but also BHA, VC and edaravone have been reported to cross the blood-brain barrier, although their degree of brain distribution is different [24]–[29]. In addition, edaravone is a drug expected to combat ROS-related diseases in the brain [35]. It is, therefore, unlikely that the failure of BHA, VC and edaravone to protect against a reduction in gonadotropins is due to their poor distribution to the fetal brain.

As only LA out of the four anti-oxidants was an effective protector, it is also suggested that LA protects the fetal expression of gonadotropins from TCDD by a mechanism different from its anti-oxidative effect. This was supported by the observation that although treatment of pregnant rats with TCDD (1 µg/kg, GD15) tends to enhance TBARS content in the fetal hypothalamus (GD20), LA failed to counteract it (Figure S4). LA itself exhibited the trend of oxidative stress enhancer, as far as the effect on the fetal hypothalamus is concerned (Figure S4). LA competes with oxidative stress not only by scavenging ROS, but also by inhibiting the ROS-mediated activation of nuclear factor kappa B (NF-κB) which is a key regulator for a number of genes [33], [34]. However, the inhibitory effect on NF-κB activation is not specific to LA, and the same is seen for edaravone [36], which has no protective effect against a TCDD-mediated reduction in fetal gonadotropins. LA also enhances the expression of nuclear factor-erythroid 2-related factor 2 (Nrf2) [37], a key regulator for a number of anti-oxidative genes [38]. Indeed, when we administered LA to pregnant rats, a significant increase in pituitary Nrf2 mRNA was observed in their fetuses at GD20 [2.32±0.56 compared with the control ( = 1.0)]. However, a similar increase was also observed following treatment of pregnant rats with BHA [3.90±0.24 compared with the control ( = 1.0)], which is ineffective against the TCDD-produced damage to fetal gonadotropins. The above pieces of evidence disagree with a view that a reduction in oxidative stress by modulating NF-κB and Nrf2 expression is a major mechanism explaining the protective effect of LA on the impaired expression of fetal gonadotropins.

It is of interest whether LA exerts its protective effect against TCDD toxicity by eliminating AhR activation. In the present study, neither TCDD-mediated induction of hypothalamic nor pituitary CYP1A1 in fetuses was antagonized by LA, whereas the same dose of LA had a positive effect on the defects in gonadotropin formation. This observation suggests that a reduction in gonadotropins by TCDD occurs through a mechanism(s) not involving AhR activation. However, this conclusion cannot be accepted until more careful studies examine other possibilities. For example, LA may target an event(s) taking place after AhR activation to restore the damage to gonadotropin synthesis. The present study also provided evidence that LA is ineffective against the acute toxicity of TCDD in pubertal rats as well as the induction of hepatic CYP1A1. Because of the difference in LA effect between defects in fetal steroidogenesis and acute toxicity, these toxic effects appear to take place via different mechanisms.

Besides its anti-oxidative nature, LA serves as a co-factor in internal metabolic reactions. For example, LA is needed in the following reactions for internal metabolism shown in parentheses: acetyl-CoA synthesis from pyruvic acid (energy production), acyl-CoA synthesis (fatty acid metabolism), conversion of α-ketoglutaric acid to succinyl-CoA (TCA cycle), and glycine cleavage (nucleotide synthesis) [34]. Although *de novo* synthesis may supply sufficient LA needed for intermediary metabolism [39], it is one possibility that fetuses and/or infants are more sensitive than adults to the impairment of LA-dependent metabolism. Indeed, knocking out the LA synthase gene in mice is fatal to the fetuses [40]. Therefore, it would be reasonable to consider that LA protects the fetal synthesis of gonadotropins from TCDD through its characteristic action as a co-factor for physiological reactions. On the other hand, pituitary synthesis of gonadotropins requires stimulation by gonadotropin-releasing hormone (GnRH) which is secreted from particular neurons projected by way of the hypothalamus [41]. As discussed before, the correct differentiation and development of fetuses appear to need stimuli by their own gonadotropins. To achieve this, the emergence and maturation of GnRH neurons would be critically important. Our previous studies have shown that maternal exposure to TCDD does not affect the fetal content of hypothalamic GnRH at GD20 [13]. However, this observation would not allow us to exclude the possibility that TCDD affects either the projection of GnRH neurons to appropriate sites or the release of GnRH to reduce the synthesis of pituitary gonadotropins.

Accumulating evidence suggests that the proper maturation or action of GnRH neurons need multiple hypothalamic factors including a sufficient supply of energy, the supply of effectors from other neurons projecting to GnRH neurons, and the activation of astrocytes [42]–[45]. In relation to this, the present study provided evidence that TCDD affects the fetal metabolome more markedly in the hypothalamus than in the pituitary. The effect of TCDD on the TCA cycle and related metabolism of amino acids in the fetal hypothalamus is schematically summarized in Fig. 8. This study strongly suggests that TCDD suppresses acetyl-CoA and succinyl-CoA production both of which need LA as a co-factor. The marked accumulation of oxaloacetic acid and α-ketoglutaric acid, and a concomitant reduction in citric acid and succinic/fumaric acid agree well with the above mechanism (see also Table 2). It is of great interest how TCDD suppresses the TCA cycle. In this context, TCDD reduced the total content of LA and DHLA in the fetal hypothalamus. This result demonstrates that TCDD suppresses the TCA cycle by reducing LA production, and it is reversed by supplying exogenous LA. Our current DNA microarray assay has suggested that the expression level of fetal hypothalamic enzymes involved in the TCA cycle and LA production remains unchanged even following TCDD treatment (refer to GSE32459 by NCBI for details). In addition, no change by TCDD in TCA cycle-regulating machinery such as pyruvate dehydrogenase kinase could be detected. Thus, TCDD seems to exert its effect on TCA cycle and LA synthesis by either the modification of the function of enzymes/regulators or the modification of the expression of regulators working at the distal steps.

*N*-Acetylaspartic acid is a nervous system-specific metabolite, and plays an important role in energy production in the brain by serving as an acetyl-CoA precursor [47]. In the central nervous system, α-ketoglutaric acid is favorably produced by aspartate aminotransferase which transfers the amino group of glutamic acid to oxaloacetic acid, yielding aspartic acid [48]. Therefore, a concomitant TCDD-induced increase in *N*-acetylaspartic acid and α-ketoglutaric acid seems reasonable. Since LA rectifies all TCDD damage to fetal steroidogenesis, gonadotropin formation and energy-producing systems, it is likely, at least in part, that TCDD impairs fetal differentiation and development by initially damaging the hypothalamic metabolome. LA also has the ability to suppress the activation of hypothalamic AMP-activated protein kinase which is induced by depletion of cellular energy [49]. Since the activation of this kinase leads to the extinguishing of GnRH neuron firing [50], it is possible that LA suppresses the activation of the above kinase directly or by improving the energy status to maintain the action of GnRH neurons. The masculine differentiation of the preoptic area, one of the hypothalamic regions playing a central role for sexual behaviors, also needs the cross-talk among GnRH neuron and other neurons which secrete effectors such as GABA and glutamic acid [44], [45]. Regarding this issue, maternal exposure to TCDD reduced the hypothalamic contents of GABA and glutamine in fetuses (see Table 1). Therefore, TCDD may disturb the development or function of GnRH neuron by reducing above neurotransmitters. LA facilitates glutamate release from nervous terminals [51]. This observation would agree with a possibility that LA competes with a TCDD-induced reduction in the pituitary expression of gonadotropins by recovering the contents of hypothalamic neurotransmitters. On the other hand, we have recently reported that TCDD reduces the fetal expression of LH via a reduction in the acetylation of histone twisted around LH gene promoter [52]. Regarding this finding, another recent study has demonstrated that the status of histone acetylation in the brain is affected by energy status [53]. Taken together, TCDD is suggested to suppress the fetal expression of LH through affecting the metabolome serving on energy production and/or signal transduction.

When LA is given orally to animals and humans, it is converted to a number of metabolites by first-pass metabolism [54]. Therefore, in this pilot study, we used an intravenous injection to examine the effect of LA. Although a wide variety of beneficial effects are expected for dietary LA [39], future studies are needed to clarify whether this substance given orally can restore the TCDD-reduced expression of fetal gonadotropins.

In conclusion, the present study demonstrates that a change in the hypothalamic metabolome plays an important role in the damage by TCDD to the fetal pituitary-gonad axis and sexual maturation. Since steroidogenesis during the 'critical period' from the late fetal to infant stages is essential for brain/sex differentiation, how to protect fetuses/infants from dioxin-induced damage to steroidogenesis is a rather important issue. If we expect an inhibitory agent to achieve this, its safety must be guaranteed. In this context, LA is a tissue constituent, and a wide variety of beneficial effects are expected from dietary LA. On the basis of the present study, we may be able to construct a dietary strategy to protect later generations from dioxin.

## Supporting Information

Figure S1 **The LA-specific recovery from a TCDD-induced reduction in the fetal expression of testiclular CYP11A1 (A) and 3β-hydroxysteroid dehydrogenase (HSD)(B) mRNAs.** Each mRNA in the fetal (GD20) testis was analyzed by RT-PCR after maternal exposure to TCDD (GD15) and anti-oxidants (GD15-20), and normalized by the intensity of β-actin cDNA. See Materials and Methods for the details of animal treatment. In the white bar control, pregnant rats were treated with DMSO alone or anti-oxidant dissolved in DMSO. In the shaded bar control, dams were given aqueous NaCl alone or anti-oxidant dissolved in this solution. Each bar represents the mean value relative to the control ± SEM. The values of two fetuses from one dam were averaged to become one analytical unit. Thus, the data are shown as n = 5 dams. *p<0.05 and **p<0.01, compared with the respective controls.
(TIF)

Figure S2 **The effect of maternal co-treatment with TCDD and anti-oxidants on the fetal expression of pituitary prolactin.** Prolactin mRNA in the fetuses (GD20), the parents of which were treated with TCDD (GD15) and anti-oxidants (GD15-20), was determined. The hormone cDNA was normalized by β-actin cDNA. The pituitaries of all male fetuses in one dam were pooled to become one analytical unit. See the legend to Figure S1 for symbol explanation. Each bar represents the mean value relative to the control ± SEM of 5 samples prepared from different dams. No significant difference was observed between the control and TCDD-treated groups.
(TIF)

Figure S3 **Principal component analysis (PCA) regarding TCDD effect on the fetal pituitary and hypothalamic metabolome: the data from positive ion mode analysis. A and B, TCDD effect on the profile of pituitary (A) and hypothalamic (B) metabolome.** Each dot is different individual (n = 16). C and D, Fragment ions in LC-TOF-MS analysis that exhibit an alteration by TCDD treatment in the pituitary (C) and hypothalamus (D). Each dot shows a single ion with a particular mass (*m/z*). The criteria for selecting ions which were significantly changed by TCDD was set either at more than 0.8 or less than −0.8 of the correlation coefficient. See Materials and Methods for details.
(TIF)

Figure S4 **The effect of maternal co-treatment with TCDD and LA on the TBARS content of the fetal hypothalamus.** TBARS content in the hypothalamus of fetuses (GD20), the parents of which were treated with TCDD (GD15) and LA (GD15-20), was determined. The hypothalami of all male fetuses in one dam were pooled to become one analytical unit. Each bar represents the mean value relative to the control ± SEM of 5 samples prepared from different dams. No significant difference was observed between the control and any other groups.
(TIF)

Methods S1 **Supplemental Methods including Supplemental Literatures.**
(DOC)
