# Supplementary figures and images for: Restoration of Dioxin-Induced Damage to Fetal Steroidogenesis and Gonadotropin Formation by Maternal Co-Treatment with α-Lipoic Acid
Source: PLoS One. 2012 Jul 20;7(7):e40322. doi: 10.1371/journal.pone.0040322 (PMC3401201; doi:10.1371/journal.pone.0040322)

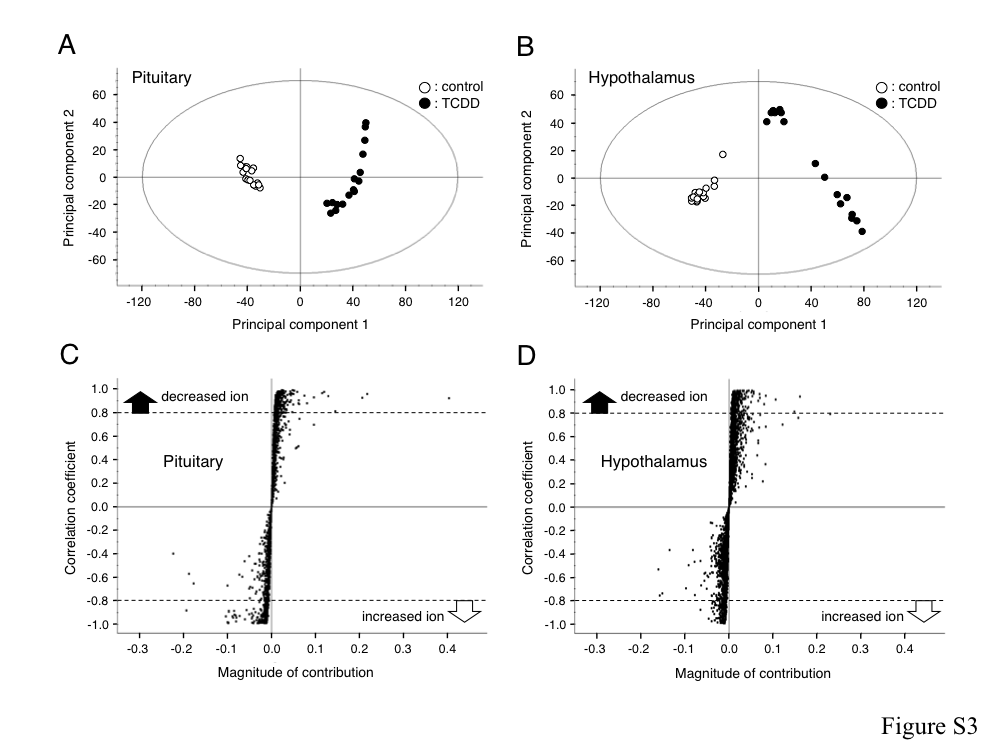

Supplement: Figure S3 — Principal component analysis (PCA) regarding TCDD effect on the fetal pituitary and hypothalamic metabolome: the data from positive ion mode analysis. A and B, TCDD effect on the profile of pituitary (A) and hypothalamic (B) metabolome. Each dot is different individual (n = 16). C and D, Fragment ions in LC-TOF-MS analysis that exhibit an alteration by TCDD treatment in the pituitary (C) and hypothalamus (D). Each dot shows a single ion with a particular mass (m/z). The criteria for selecting ions which were significantly changed by TCDD was set either at more than 0.8 or less than −0.8 of the correlation coefficient. See Materials and Methods for details. (TIF) [file pone.0040322.s003.tif]
