# Supplementary material for: Restoration of Dioxin-Induced Damage to Fetal Steroidogenesis and Gonadotropin Formation by Maternal Co-Treatment with α-Lipoic Acid
Source: PLoS One. 2012 Jul 20;7(7):e40322. doi: 10.1371/journal.pone.0040322 (PMC3401201; doi:10.1371/journal.pone.0040322)
Supplement: Methods S1 — Supplemental Methods including Supplemental Literatures. (DOC) [file pone.0040322.s005.doc]

**Methods S1**

Metabolomics and analysis of tricarboxylic acid (TCA) cycle components

The extraction of polar metabolites was conducted by a reported method with some modifications (1, 2). The male fetal hypothalami and pituitaries in one dam were separately pooled, and homogenized with 3.5-volume of cold methanol (350 μl/100 mg). After spiking with 300 ng glycylnorleucine as an internal standard (I.S.), the homogenate was mixed vigorously with an equal volume of water, and then with 3 volumes of chloroform based on the tissue weight. After centrifugation, the upper phase was washed with the same volume of chloroform, and dried in a vacuum concentrator. For the metabolomics, the extracts dissolved in 0.1 ml 15% acetonitrile were loaded onto a Varian C18 cartridge to remove peptides and proteins (3). The cartridge was washed with 0.8 ml 15% acetonitrile, and the pass-through fraction was dried *in vacuo*. The residue was reconstituted in 50 μl acetonitrile, and an aliquot (10 μl) was subjected to LC-TOF-MS analysis. To determine the constituents of the TCA cycle, calibration curves were made after adding standards to the hypothalamus homogenate together with an I.S., glycylnorleucine. In this case, the signal of the endogenous component detected in the blank was subtracted. The molecular mass used for detection and the retention times of the analytes were as follows: citric acid, *m/z* 191.0192 ([M-H] -) and 1.17 min; α-ketoglutaric acid, *m/z* 145.0137 ([M-H] -) and 1.19 min; *cis*-aconitic acid, *m/z* 173.0086 ([M-H]^-^) and 1.23 min; fumaric acid, *m/z* 115.0031 ([M-H] -) and 1.33 min; malic acid, *m/z* 133.0137 ([M-H] - ) and 1.34 min; succinic acid, *m/z* 117.0188 ([M-H] -) and 1.40 min; isocitric acid, *m/z* 191.0192 ([M-H] -) and 2.04 min; oxaloacetic acid, *m/z* 130.9980 ([M-H] -) and 2.67 min; and glycylnorleucine (I.S.), *m/z* 187.1083 ([M-H] -) and 10.47 min.

Determination of LA and DHLA

The extraction of LA/DHLA was conducted by a reported method with some modifications (4). The male fetal hypothalami in one dam were separately pooled, and homogenized with an equal volume of cold water. After spiking with 300 ng sodium valproate as an internal standard (I.S., 6 ng/μl ethanol), the homogenate was mixed vigorously with 6 volumes of acetonitrile relative to the tissue weight. After centrifugation, the upper phase was collected. This step was repeated one more time. The upper phases were pooled, and dried in a vacuum concentrator. The residue was reconstituted in 50 μl of 40% acetonitrile (v/v), and an aliquot (10 μl) was subjected to LC-TOF-MS analysis. To determine the content of LA/DHLA, calibration curves were made after adding standards to the hypothalamus homogenate together with an I.S. The molecular mass used for detection and the retention times of the analytes were as follows: LA, *m/z* 205.0357 ([M-H] -) and 2.42 min; DHLA, *m/z* 207.0513 ([M-H] -) and 2.40 min and sodium valproate (I.S.), *m/z* 142.0994 ([M-H-Na] -) and 1.18 min.

LC-TOF-MS conditions

An ACQUITY UPLC system equipped with a TOF-MS, SYNAPT G2-S (Waters, Manchester U.K.) was used. In the quantification of testosterone, the operating conditions of LC were as follows: column, BEH C18 column (1.7 μm particle size, 2.1 × 100 mm, Waters); column temperature, 40°C; sample room temperature, 10°C; mobile phase, changing acetonitrile ratio (%, v/v) in 0.1% (v/v) formic acid as follows: 10% (0-3 min), 10 to 100% (3-18 min), 100% (18-20 min) and then stepwise returning to 10% and maintaining this for 3 min; flow rate, 0.2 ml/min. In the metabolmics and the determination of TCA cycle components, the samples were analyzed under the following conditions: Column, HSS T3 column (1.8 μm particle size, 2.1 × 100 mm, Waters); column temperature, 40°C; sample room temperature, 4°C; mobile phase, changing the acetonitrile ratio (%, v/v) in either 0.1% (v/v) formic acid (for the analysis of positive ions) or 10 mM 4-methylmorpholine (for the analysis of negative ions) as follows: 2% (0-8 min), 2 to 100% (8-16 min), 100% (16-18 min) and then stepwise returning to 2% which was maintained for 5 min; flow rate, 0.2 ml/min. In the quantification of LA and DHLA, the samples were analyzed under the following conditions: Column, HSS T3 column (1.8 μm particle size, 2.1 × 100 mm, Waters); column temperature, 40°C; sample room temperature, 4°C; mobile phase, changing the acetonitrile ratio (%, v/v) in 0.1% (v/v) acetic acid (pH4, adjusting with aqueous ammonia) as follows: 40 to 95% (0-1.9 min), 95% (1.9-2.5 min), stepwise returning to 40%, and maintaining this for 4.5 min; flow rate, 0.2 ml/min.

As for the conditions of TOF-MS in testosterone analysis, ionization was achieved by electro-spray ionization. The capillary voltage was set at 3.5 kV in positive ion mode. The cone and multiplier voltages were set at 50 and 550 V, respectively. Leucine enkephaline (200 pg/ml 50% methanol containing 0.1% formic acid) was used as the I.S. for lockmass-correction. This solution was infused at a rate of 5 μl/min via a lockspray interface (baffling frequency, 0.5 s^−1^), and the instrument was adjusted so that the molecular mass of the ion m/z was 557.2802. The source temperature was 120°C, and desolvation was performed at 350°C with a nitrogen flow rate of 600 l/hr. The mass spectrometer was tuned to 9,000 mass resolution, and spectra were recorded by detecting ions ranging from m/z 50 to 300. The same conditions were used for the metabolomics and the analysis of TCA cycle components, LA and DHLA content, except that the capillary voltage was set at 3.0 kV, and the instrument was adjusted to give a molecular mass of I.S. at m/z 555.2648 in negative ion mode. While only negative ion mode was used in the analysis of TCA cycle components and LA/DHLA content, both positive and negative ion modes were used for metabolomics.

Multivariate analysis

Metabolome profiles were compared with each other using principal component analysis (PCA), one form of multivariate analysis, which is capable of distinguishing several groups containing a number of variables (5). The information including the retention times and intensity of ions obtained by LC-TOF-MS was normalized by the internal standards and tissue weights used, and converted to a data matrix capable of being compared with data from other samples using MarkerLynx^TM^XS software (Waters). The data matrix from different rats was subjected to PCA, using the same software. To compare the metabolome profile between the control and TCDD-treated group in the hypothalamus and pituitary, these data were analyzed using an orthogonal partial least-squares to latent structures-discriminant analysis (OPLS-DA) method using a combination of MarkerLynx^TM^XS and SIMCA-P software (MKS-Umetrics, Malmo, Sweden). From the OPLS-DA score obtained, fragment ions that contributed to the TCDD-dependent alteration were plotted (S-plot). On this basis, cellular ingredients that contribute to TCDD-produced changes were identified by selecting compounds making a contribution to the correlation co-efficient which was either more than +0.8 or less than -0.8. The compounds detected above were identified by referring the retention time and mass information to online databases, such as the Human Metabolome Database (http://www.hmdb.ca/) and the Kyoto Encyclopedia of Genes and Genomes (KEGG, http://www.genome.jp/).

**Supplemental Literatures**

1. Le Belle JE, Harris NG, Williams SR, Bhakoo KK (2002) A comparison of cell and tissue extraction techniques using high-resolution ^1^H-NMR spectroscopy. NMR Biomed 15: 37-44.

2. Sun G, Yang K, Zhao Z, Guan S, Han X, et al. (2002) Shotgun metabolomics approach for the analysis of negatively charged water-soluble cellular metabolites from mouse heart tissue. Anal Chem 79: 6629-6640.

3. Sari Y, Hammad LA, Saleh MM, Rebec GV, Mechref Y (2010) Alteration of selective neurotransmitters in fetal brains of prenatally alcohol-treated C57BL/6 mice: quantitative analysis using liquid chromatography/tandem mass spectrometry. Intl J Dev Neurosci 28: 263-269.

4. Cheng HT, New LS, Neo AH, Goh CW, Browne ER, Chan EC (2009) Distribution study of orally administered lipoic acid in rat brain tissues. Brain Res 1251:80-86.

5. Trygg J, Holmes E, Lundstedt T (2007) Chemometrics in metabonomics. J Proteome Res 6: 469-479.
